# Effects of dietary l-carnosine supplementation on the growth, intestinal microbiota, and serum metabolome of fattening lambs

**DOI:** 10.3389/fvets.2024.1525783

**Published:** 2025-01-22

**Authors:** Yaxuan Meng, Tingting Xian, Guolei Kang, Hongna Wang, Tao Feng

**Affiliations:** ^1^Institute of Animal Husbandry and Veterinary Medicine (IAHVM), Beijing Academy of Agriculture and Forestry Sciences (BAAFS), Beijing, China; ^2^College of Animal Science and Technology, Gansu Agricultural University, Lanzhou, Gansu, China; ^3^Joint Laboratory of Animal Science Between IAHVM of BAAFS, Division of Agricultural Science and Natural Resource of Oklahoma State University, Beijing, China; ^4^College of Life Science and Food Engineering, Hebei University of Engineering, Handan, China

**Keywords:** fattening lamb, L-carnosine, microbiota, metabolomics, multi-omics analysis

## Abstract

Dietary l-carnosine supplementation has been shown to enhance animal performance and improve meat quality. However, the mechanisms underlying the effects of l-carnosine on the physiological functions of animals have not been fully elucidated. We investigated the effects of dietary l-carnosine supplementation on growth performance, intestinal microbiota diversity, and the serum metabolome in fattening lambs to reveal the molecular mechanism underlying the effect of l-carnosine on the growth performance of sheep. Sixty 3-month-old male crossbred lambs (Dorper ♂ × Small Tail Han ♀) with an average body weight of 30 ± 5 kg were randomly divided into two groups: a control group (group C) fed a basal diet, and an experimental group (group L) fed a basal diet supplemented with 400 mg/kg of l-carnosine. At the end of the 60-day experiment, all sheep were weighed, and fecal and blood samples were collected from 12 random sheep. The fecal microbiota was analyzed using 16S rRNA sequencing, and serum metabolites were analyzed using liquid chromatography–tandem mass spectrometry. Spearman correlation analysis was employed to assess the associations between intestinal microbiota and serum metabolite biomarkers. The results showed that weight gain and daily weight gain were significantly increased in group L compared to group C (*p* < 0.01). The dominant phyla in the intestinal microbiota (Firmicutes and Bacteroidetes) did not significantly differ between the two groups (*p* > 0.05). At the genus level, the abundances of *Syntrophococcus* (*p* < 0.01) and *Butyricimonas* (*p* < 0.001) were higher, whereas those of *Escherichia-Shigella* and *Candidatus Saccharimonas* were significantly lower in group L than in group C (*p* < 0.05). Non-targeted metabolomics identified 68 differentially abundant biomarkers (VIP > 1, *p* < 0.05). The content of pyridine N-oxide glucuronide was significantly downregulated (*p* < 0.01), whereas those of l-histidinol, d-apiose, and isodomedin were significantly upregulated in group L versus group C (*p* < 0.001). *Holdemania* and *Butyricimonas* were positively correlated with l-histidine, d-apiose, and l-erythrulose (*p* < 0.001), whereas *Butyricimonas* was negatively correlated with pyridine N-oxide glucuronide (*p* < 0.001). This study provided new insights into the effects of l-carnosine on the intestinal microbiota and nutrient metabolism in fattening sheep that will be helpful for the future application of l-carnosine in ruminants.

## 1 Introduction

With the enhancement of human living standards, the demand for livestock products is increasing, which is an excellent opportunity for animal husbandry, including the sheep farming industry (1). Carnosine is a dipeptide found in animals (2), but its content varies among species and animal body parts (3, 4). Carnosine and l-carnosine have the same chemical structure and biological activity, and as the amino acid residues in carnosine have an l-form stereochemical configuration, it can also be referred to as l-carnosine. l-Carnosine is present at high concentrations in the skeletal muscle of most vertebrates and has critical antioxidant functions (5). In porcine myoblasts, it promoted cell proliferation by activating targets of the mammalian the rapamycin signaling pathway and mitigated cellular damage caused by oxidative stress (6). In mice exposed to deoxynivalenol, l-carnosine bound to Keap1, releasing the transcription factor Nrf2 into the nucleus, thereby activating the transcription of downstream genes and increasing antioxidant production to neutralize excess reactive oxygen species and thus reduce the oxidative stress caused by deoxynivalenol (7). Further, l-carnosine helps maintain animal health through anti-glycosylation (8, 9), pH stabilization, and metal chelation (10). Studies have shown that l-carnosine ameliorates the adverse effects of oxidative stress in pregnant ewes, and it may also offer benefits in reducing the combined challenges posed by pregnancy and heat stress during the hot-dry season (11). In finishing pigs, dietary l-carnosine increased body weight, feed intake, and daily gain, increased the secretion of thyroid hormone, and induced the proliferation of satellite cells (12). In chickens, l-carnosine also positively influences weight gain and activates the enzymatic antioxidant system in the blood (13, 14).

The intestinal microbiota plays a vital role in adaptive coevolution with mammals (15). The intestinal tract of sheep is characterized by a diverse microbial ecosystem (16), which is influenced by multiple factors, such as feeding, drinking, the environment, physiology, and disease (17–19). The intestinal microbiota also plays a crucial role in biological processes related to regulating nutrient absorption and maintaining homeostasis (20). It produces various digestive enzymes that convert indigestible plant macromolecules into small molecules that are absorbable by the host (21). For example, certain microorganisms produce pectinases that can degrade pectin in the plant cell wall into small molecules that can be utilized by ruminants (22), whereas many species produce lipases that hydrolyze long-chain fatty acids (23). Once absorbed by the host, these simple metabolites enter the bloodstream, altering serum metabolite levels (24). Microbial homeostasis influences the efficiency with which the host animal utilizes its feed, ultimately affecting productive performance metrics, such as daily weight gain and the feed conversion rate.

Metabolomics plays a crucial role in analyzing metabolic pathways, processes, and gene functions (25, 26), and is widely used in the fields of animal disease diagnosis and food ingredient identification (27). Comprehensive analysis of the correlations between the intestinal microbiota and serum metabolome not only enables identifying the relationship between dietary energy levels and lamb quality (28) but has also been used to investigate how they reflect age and nutritional requirements in Tibetan sheep (29). Furthermore, it has well explained the effects of different diets on intestinal microbes and metabolic pathways in Hu sheep (30).

Our knowledge about the effects of l-carnosine on the intestinal microbiota and serum metabolome in fattening sheep is limited, and published studies are scarce. We investigated the effects of dietary l-carnosine supplementation on the fecal microbiota and metabolite composition in fattening lambs using 16S rRNA sequencing and liquid chromatography–tandem mass spectrometry (LC–MS/MS)-based metabolomics. Additionally, multi-omics analysis was conducted on the fecal microbiota and serum metabolites to provide a reference for the application of l-carnosine in sheep.

## 2 Materials and methods

### 2.1 Experimental animals and experimental design

The experiment was conducted at the Sheep Experimental Station of the Beijing Academy of Agriculture and Forestry Sciences in Yangyuan County, Zhangjiakou City, Hebei Province, China. In total, 60 male crossbred lambs (Dorper ♂ × Small Tail Han ♀) of 3 months of age with an average body weight of 30 ± 5 kg were randomly divided into two groups. The lambs were housed in 2 sheltered outdoor paddocks and fed a basal diet consisting of a total mixed ration according to the Chinese sheep feeding standard (NY/T816-2004). The contents of digestible energy, metabolizable energy, crude protein, calcium, and phosphorus in the diet were 11.83 MJ·kg-1, 9.73 MJ·kg-1, 14.61, 0.39, and 0.25%, respectively (31). Animals in the control group (group C) were fed only the basal diet (Table 1). Based on previous studies and the dietary supplementation level of l-carnosine used previously in fattening pigs (12, 32), we determined the optimal supplementation level of l-carnosine for fattening lambs to be 400 mg/kg. In the experimental l-carnosine group (group L), l-carnosine (purity ≥98%, Zhengzhou Luyuan Biotechnology Co., Ltd.) was added to the basal diet at a concentration of 400 mg/kg. Both groups were provided with adequate water and salt blocks during the 60-day trial period.

**Table 1:** Composition and nutrient levels of the basic diet of fattening sheep.

Item	Content
Ingredients	
Corn silage	30.90
Corn	44.20
Soybean meal	17.00
Wheat bran	5.00
Salt	0.70
Limestone powder	1.00
Calcium hydrogen phosphate	0.20
Premix^1^	1.00
Total	100.00
Nutrientcomponent^2^	
Metabolizable energy	9.73
Digestible energy	11.83
Crude protein	14.61
Calcium	0.39
Phosphorus	0.25

^1^The premix provided the following per kg diets: VA 200 kIU, VD_3_ 40 kIU, VE 1000 mg, Nicotinamide 1,200 mg, Biotin 70 mg, Fe (FeSO4) 1300 mg, Mn (MnSO4) 1000 mg, Zn (ZnSO4) 1240 mg, Se (Na2SeO3) 8.50 mg, Co (CoSO4) 11 mg. ^2^Metabolizable energy was a calculated value, while the others were measured values.

### 2.2 Sample collection

The morning after the feeding experiment, the 60 sheep were weighed. Fresh feces were individually collected from the rectum of 12 random sheep in each group (*n* = 24 in total), immediately transferred into sterile tubes, and frozen in liquid nitrogen. To prevent contamination, the outer layer of each fecal sample was discarded, and the middle inner portion was sampled for testing. Blood (5 mL) was collected from the jugular veins of the above 24 sheep using vacuum blood collection tubes for serum metabolomics analysis. The blood samples were maintained at room temperature for 4 h, followed by centrifugation at 3000 rpm, 4°C for 10 min to collect the serum. The serum was frozen at −80°C until metabolomics analysis.

### 2.3 Microbial DNA extraction and sequencing

Microbial DNA was extracted from the fecal using an E.Z.N.A.® Soil DNA Kit (Omega Bio-tek, Norcross, GA,U.S.). The DNA was examined using 2% agarose gel electrophoresis, and the DNA concentration and purity were determined with a Thermo Fisher NanoDrop-2000 spectrophotometer (Thermo Scientific, U.S). The variable regions V3 and V4 of the bacterial 16S rRNA gene were PCR-amplified using primers 338F (5′-ACTCCTACGGGGAGGCAGCAG-3′) and 806R (5′-GGACTACHVGGGGTWTCTAAT-3′). The PCR products were recovered, purified, assayed, and quantified using a microfluorometer (QuantiFluor-ST; Promega, USA). The PCR products were mixed in equimolar amounts, and a sequencing library was constructed using a NEX-TFLEX Rapid DNA-Seq Kit and sequenced on the Illumina MiSeq PE 300 platform (Shanghai Majorbio Bio-Pharm Technology Co., Ltd.).

### 2.4 Microbiome composition analysis

Operational taxonomic units (OTUs) at a 97% similarity level were clustered and compared to the 16S rRNA database (Silva v138/16S_bacteria) to classify annotations of the OTU sequences, setting the classification confidence to 0.7. All data were analyzed on the Shanghai Majorbio Cloud platform. The alpha diversity index was determined using Mothur. Differences or similarities in the composition of the fecal microbial communities were analyzed using principal coordinates analysis (PCoA). Abundance differences in intestinal microbial composition at various levels between the two groups were analyzed using Student’s *t*-test. The linear discriminant analysis effect size (LEfSe) algorithm was used to identify differences among various taxonomic groups. Using the acquired community abundance data, rigorous statistical approaches were used to perform hypothesis testing on species across various microbial communities. This process aims to evaluate the significance of species abundance differences and pinpoint species that exhibit notable disparities between groups. PICRUSt was used to normalize the OTU abundance table to obtain the corresponding GreenGene IDs of the OTUs, and Kyoto Encyclopedia of Genes and Genomes (KEGG) functional annotations were compared against the Clusters of Orthologous Genes database to obtain annotation information of the OTUs and abundance information for each function across the samples.

### 2.5 Metabolite extraction and metabolomics analysis

For metabolite extraction, methanol was added to 100 μL of serum, after which the samples were pulverized using a cryo-mill and sonicated for 30 min (5°C, 40 kHz). The samples were left to precipitate the proteins and then centrifuged. The supernatants were transferred into sample vials for LC–MS/MS analysis.

The samples were separated by ultra-high performance liquid chromatography system (ACQUITY UPLCHSS T3, Waters Corp., USA) and detected. The raw data included quality control (QC) and detection samples. Data pre-processing for appropriate data analysis encompassed the filtering of the raw data, recoding of missing values, normalization, QC verification, and data conversion and was conducted using the Progenesis QI 2.3 software.

The MS data were matched with the metabolite database HMDB^1^ and Metlin,^2^ and data analysis was performed on the Shanghai Majorbio Cloud platform. Projected variable importance (VIP) values were calculated using orthogonal least partial squares discriminant analysis (OPLS-DA) modeling, and the validity of the OPLS-DA model was evaluated using R2Y and Q2.

Enrichment analysis was performed using Fisher’s exact test. *p*-values were corrected using the Benjamini-HochbergBH method, and the corrected *p*-values were thresholded at 0.05. KEGG pathways meeting this criterion were considered significantly enriched in the metabolite set, and the significantly enriched pathways were accurately examined using the Scipy software v1.0.0 (*p* < 0.05). The raw sequencing reads generated in this study have been deposited in the NCBI Sequence Read Archive (SRA) under BioProject accession number PRJNA1175011.^3^

### 2.6 Correlation analysis

The Spearman correlation coefficients between fecal microbial taxa and differential serum metabolites were calculated using the Python software. Correlation heatmaps were generated to visualize and analyze the associations. Spearman’s correlation analysis is a statistical technique used to assess the relationship and the strength of influence between an independent variable and a dependent variable by analyzing the rank order of two sets of variables. It involves three primary steps: first, ranking the data from both sets of variables based on their magnitude; second, replacing the original data with these ranks; and finally, calculating the correlation between these ranks. This method provides insights into the association and impact of the independent variable on the dependent variable.

## 3 Results

### 3.1 Growth performance

The effect of l-carnosine on the weight gain of fattening lambs is presented in Table 2. Total weight gain and average daily weight gain (ADG) were significantly increased, by 13.31 and 13.28%, respectively, in group L compared to group C (*p* < 0.01).

**Table 2:** Effect of l-carnosine on weight gain in fattening lambs.

Groups	Number	Initial weight/kg	Final weight/kg	Total weight gain/kg	Average daily gain/g
Group C	30	33.02 ± 2.28	47.45 ± 2.95	14.43 ± 1.79	240.56 ± 29.82
Group L	30	31.88 ± 2.65	48.25 ± 1.88	16.35 ± 1.23**	272.50 ± 20.55**

Data are presented as mean ± standard error of mean. **p < 0.01 *vs*. group C. “Group C” indicated the control group, “Group L” represented the l-carnosine of 400 mg/kg. The same as below.

### 3.2 Fecal microbiota richness and diversity

**Figure 1:**
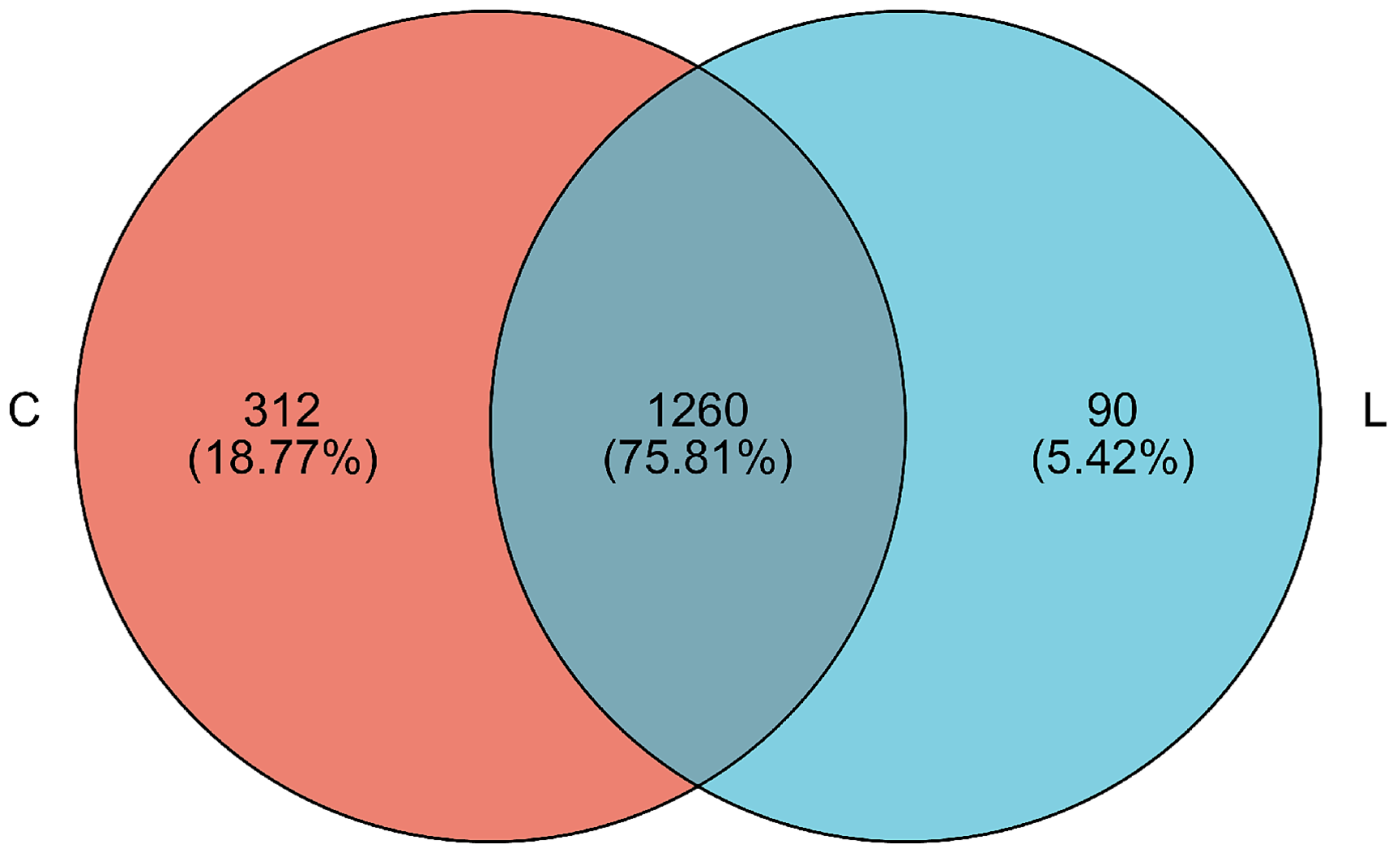
Venn diagram showing the OTUs in the fecal microbiota of fattening sheep. “C” indicated the control group, “L” represented the l-carnosine of 400 mg/kg. The same as below.

**Figure 2:**
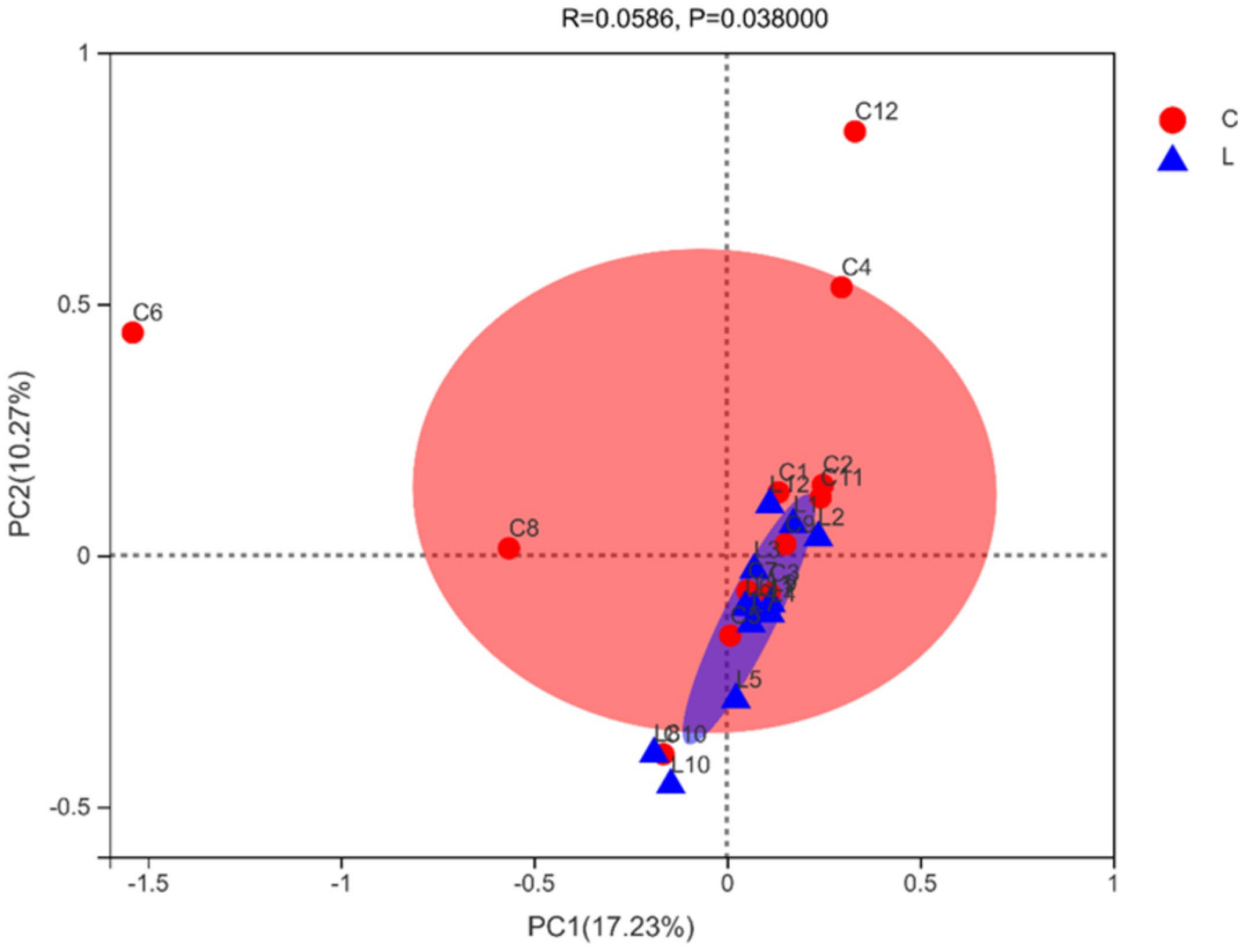
PCoA plot of microbial beta-diversity (based on the Bray–Curtis algorithm).

At the 97% similarity threshold, a total of 1,662 OTUs were found in samples from groups C and L (Figure 1). The microbiota of group C and group L samples shared 1,260 OTUs, and 312 and 90 OTUs were uniquely identified in group C and group L samples, respectively. Diversity indices were calculated based on the OTUs of each library. We assessed alpha diversity in the two groups using five indices (Shannon, Simpson, ACE, Chao, and coverage) (Table 3). No significant differences between the two groups in these indicators were found (*p* > 0.05). Beta diversity was evaluated using PCoA. A PCoA plot based on the Bray–Curtis algorithm (Figure 2) showed that groups C and group L differed in terms of beta diversity (*p* < 0.05). Notably, the group C samples exhibited a dispersed distribution in the PCoA plot, indicating substantial variation in the microbial community structures within the group. Conversely, the group L samples clustered more compactly, suggesting a higher degree of similarity in the intestinal microbial community structures among sheep supplemented with l-carnosine.

**Table 3:** Effect of l-carnosine on five alpha diversity indices related to the fecal microbiota of fattening sheep.

Groups	Shannon	Simpson	Ace	Chao	Coverage
Group C	4.60 ± 0.55	0.035 ± 0.031	765.5 ± 161.3	786.2 ± 165.5	0.9965 ± 0.0006
Group L	4.78 ± 0.45	0.024 ± 0.018	751.6 ± 129.1	766.2 ± 134.0	0.9966 ± 0.0006

### 3.3 Fecal microbiota composition

**Figure 3:**
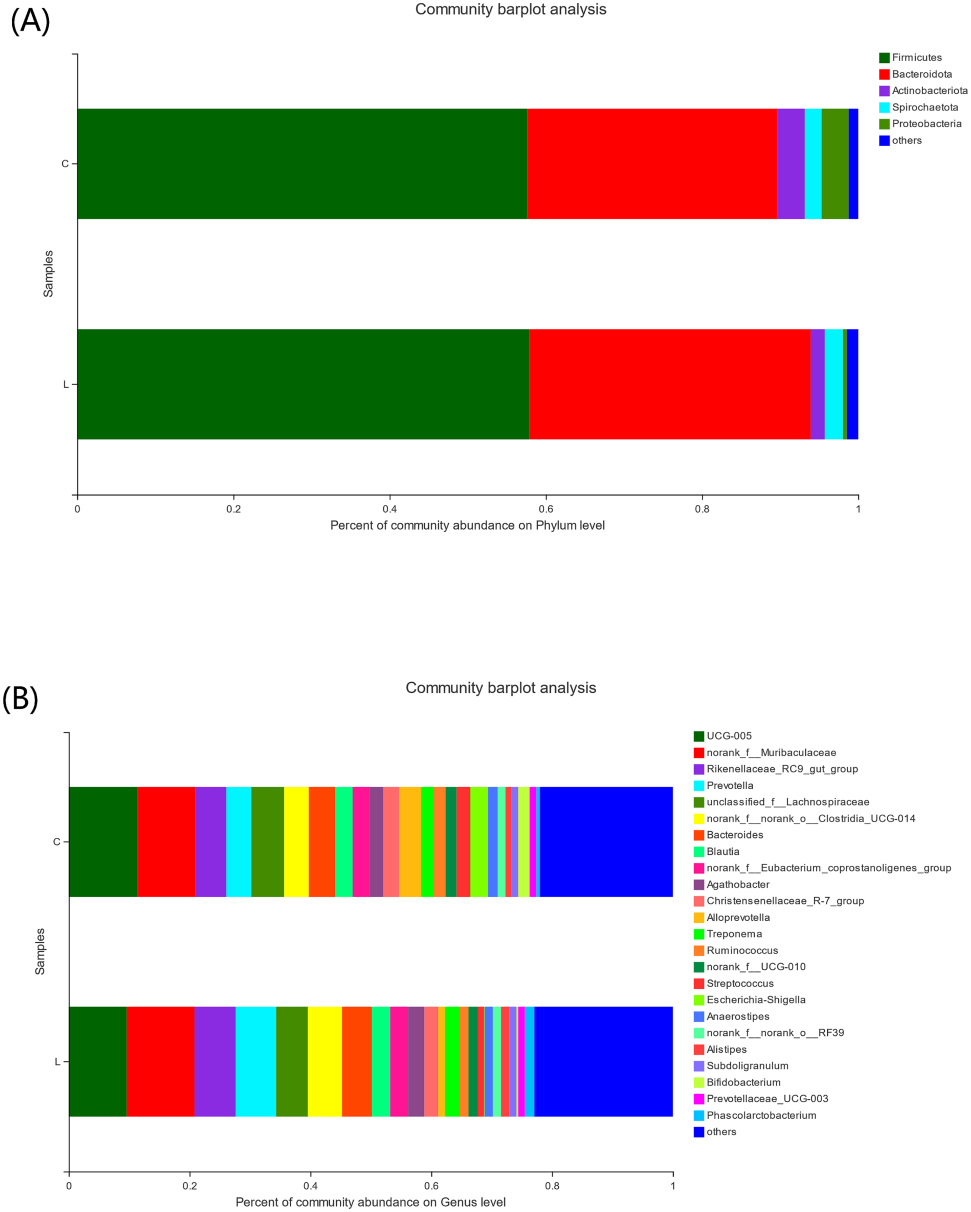
Effect of l-carnosine on fecal microbiota composition of fattening lambs. **(A)** Bacterial abundance at the phylum level. **(B)** Bacterial abundance at the genus level.

**Figure 4:**
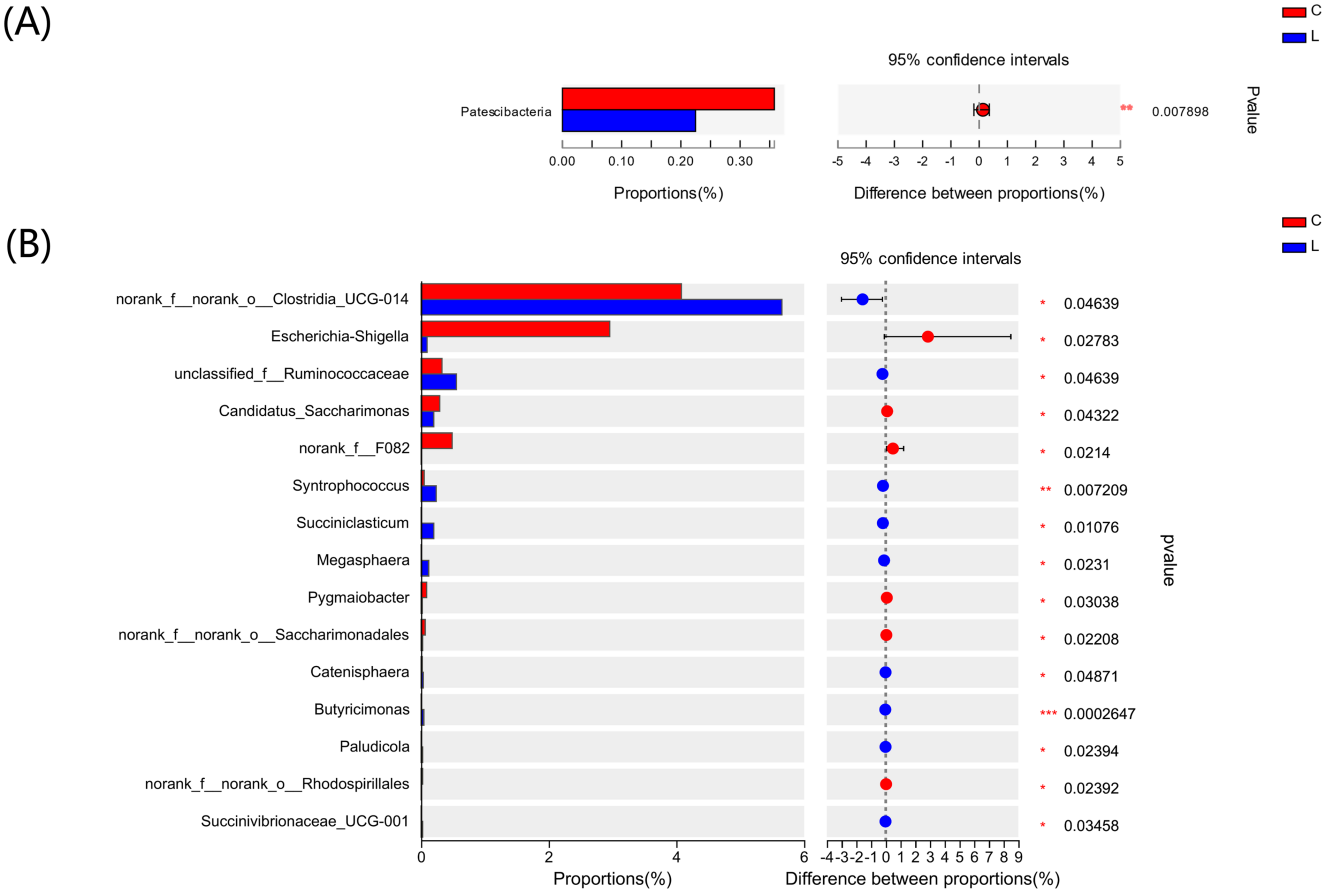
Differences in the major bacterial **(A)** phyla and **(B)** genera in lamb of control and l-carnosine group based on Wilcoxon rank-sum tests. *p < 0.05, **p < 0.01, ***p < 0.001.

To investigate the differences in the fecal microbiota composition between groups C and L, we used the Wilcoxon rank-sum test to compare the mean relative abundances of dominant bacteria at the phylum and genus levels. The dominant phyla in the two groups were Firmicutes and Bacteroidota. The abundances of Actinobacteriota and Proteobacteria were decreased, whereas those of Bacteroidota and Spirochaetota were increased in group L compared to group C (Figure 3A). The top three dominant genera in the two groups were *UCG-005*, *norank_f_Muribaculaceae*, and *Rikenellaceae_RC9_gut_group* (Figure 3B). Further analyses revealed that, at the phylum level (Figure 4A), the abundance of Patescibacteria was significantly lower in group L than in group C (*p* < 0.01). At the genus level (Figure 4B), the abundances of *Syntrophococcus* (*p* < 0.01) and *Butyricimonas* (*p* < 0.001) were higher in group L than in group C. In contrast, the abundances of *Escherichia-Shigella* and *Candidatus* Saccharimonas were significantly lower in group L than in group C (*p* < 0.05).

### 3.4 Metabolomics analysis

Using non-targeted metabolomics technology, serum samples from groups C and L were analyzed to characterize the blood metabolites in fattening lambs with or without l-carnosine supplementation. The differences between the groups were analyzed using OPLS-DA. As shown in the OPLS-DA score plot (Supplementary Figure S1A), the samples of the two groups were clearly distinguished. The values of R2Y (0.995) and Q2 (0.657) were > 0.5 (Supplementary Figure S1B), indicating that the OPLS-DA model had a good fit and good predictive ability, and that VIP values could be calculated based on the data to screen for differential marker metabolites.

**Figure 5:**
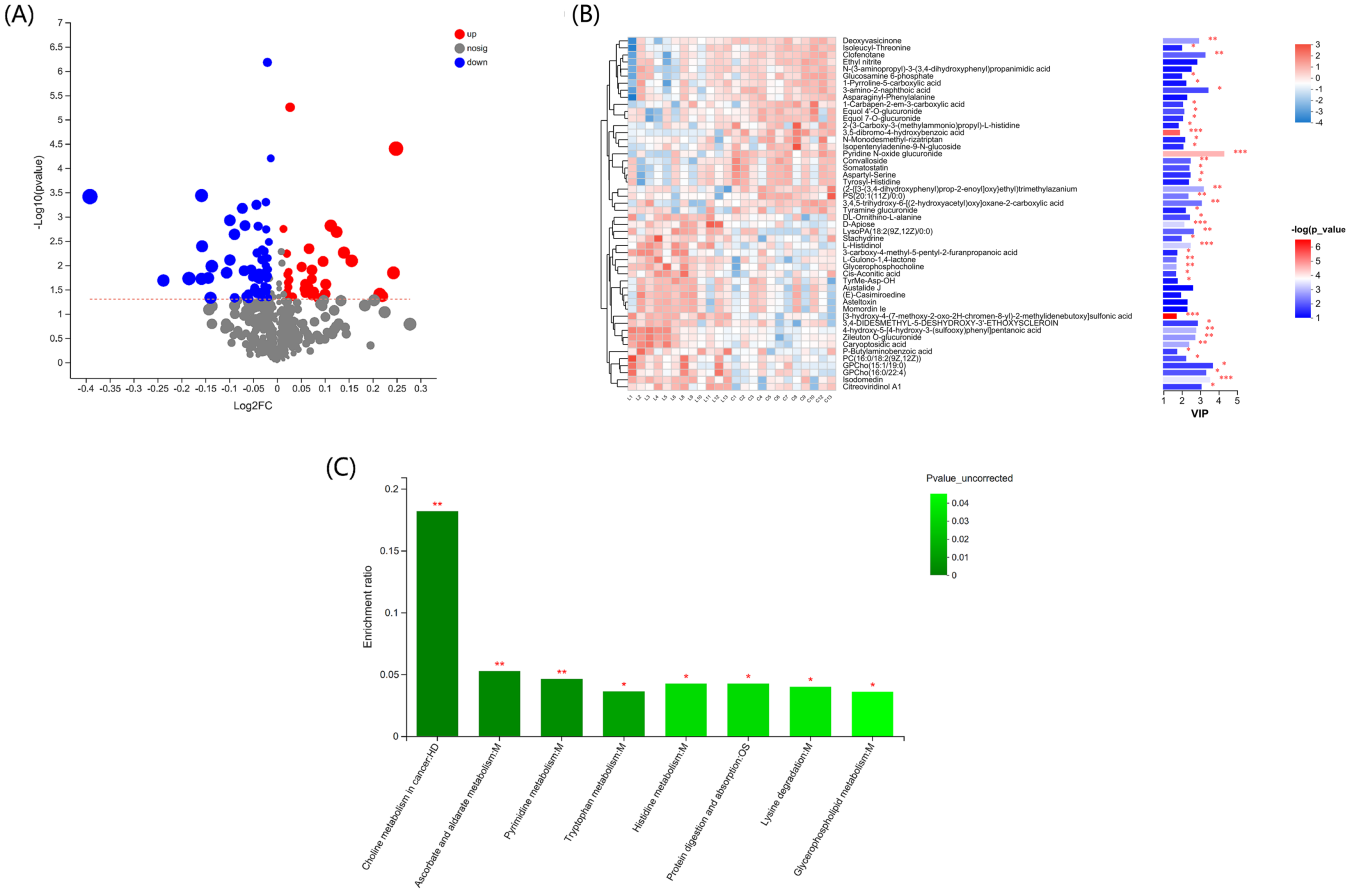
Effect of l-carnosine on the serum metabolome in fattening lambs. **(A)** Volcano plots showing the distribution of differential metabolites identified based on *p* < 0.05 and VIP > 1. **(B)** Profiling, VIP score, and *p*-values for the top 30 differential serum metabolites in groups L and C. *p < 0.05, **p < 0.01, ***p < 0.001. **(C)** KEGG metabolic pathway enrichment analysis of the differential serum metabolites in l-carnosine and control groups. *p < 0.05, **p < 0.01 vs. group C.

Using VIP > 1 and *p* < 0.05 as the optimal thresholds, 68 differential metabolites were identified (Figure 5A). The clustering heatmap of the differential metabolites shows the differential metabolite expression profiles and VIP values for the metabolites with the top 30 VIP values (Figure 5B; Supplementary Table S1). Pyridine N-oxide glucuronide was downregulated (*p* < 0.01), whereas l-histidinol, d-apiose, and isodomedin were upregulated in group L (*p* < 0.001) compared to group C.

Differential metabolites were significantly enriched in eight KEGG pathways (*p* < 0.05): choline metabolism in cancer, ascorbate and alternate metabolism, pyrimidine metabolism, tryptophan metabolism, histidine metabolism, protein digestion and absorption, lysine degradation, and glycerophospholipid metabolism (Figure 5C).

### 3.5 Correlations between fecal microbial and serum metabolites

**Figure 6:**
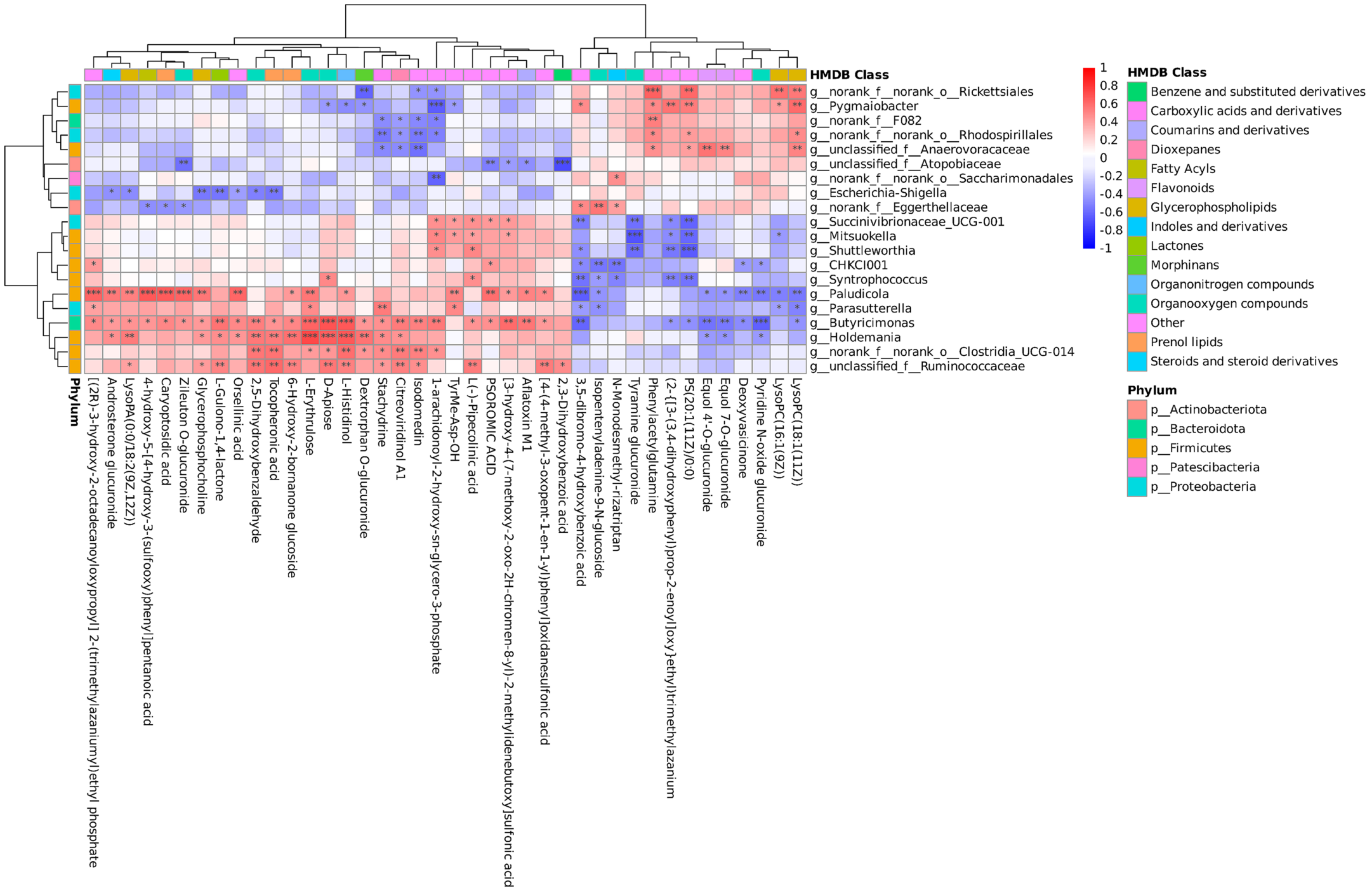
Correlation analysis of fecal microbiota and serum metabolites in fattening lambs. Red indicates a positive correlation, blue indicates a negative correlation. *p < 0.05, **p < 0.01, ***p < 0.001 indicate statistically significant correlation between fecal microbiota and serum metabolites.

The correlations between 20 genera and 40 metabolites were analyzed using Spearman correlation analysis (Figure 6). *Holdemania* and *Butyricimonas* were significantly positively correlated with l-histidinol, d-apiose, and l-erythrulose (*p* < 0.001). A highly significant negative correlation existed between *Butyricimonas* and pyridine N-oxide glucuronide (*p* < 0.001). *Pygmaiobacter* was positively correlated with phenylacetylglutamine (*p* < 0.05). A significant positive correlation was observed between *Butyricimonas* and stachydrine (*p* < 0.05).

## 4 Discussion

The dipeptide l-carnosine has been used in pig and chicken production to improve productive performance and antioxidant capacity and to maintain livestock health (33–35). However, reports on its application in sheep are scarce. Because of the unique digestive mechanism and dietary characteristics of ruminants, their intestinal microbiota is crucial for maintaining intestinal health and nutrient absorption. Intestinal microbial communities indirectly but profoundly influence the animal feed conversion efficiency and production performance by regulating the production of endogenous metabolites and various metabolic pathways (36).

The present study investigated the effects of dietary l-carnosine supplementation on the growth performance of fattening lambs. The dietary supplementation induced an increase in the total weight gain and ADG in the fattening lambs. Similar positive effects have been observed in other animal species. l-Carnosine supplementation in pigs increased the final weight, average daily feed intake, and ADG (12), and in chickens, it effectively increased body weight and breast muscle development (14). However, the mechanism by which l-carnosine is able to improve growth performance in fattening sheep remained unclear. Therefore, we analyzed the effects of l-carnosine supplementation on intestinal microbiota composition and serum metabolite profiles to gain insights into the mechanisms underlying its growth-promoting effects in fattening sheep.

The results revealed that the addition of l-carnosine did not affect the diversity and dominant species in the intestinal microbiota of fattening lambs. At the phylum level, the intestinal microbiota of fattening lambs was enriched in Firmicutes and Bacteroidetes both in the C and L groups, which is consistent with the prior findings in herbivores (37, 38). Firmicutes and Bacteroidetes are primarily responsible for carbohydrate, protein, and fiber metabolism (18, 39). At the genus level, l-carnosine supplementation significantly increased the relative abundances of *Syntrophococcus* and *Butyricimonas*. *Syntrophococcus*, a member of Firmicutes, can digest various carbohydrates and produce short-chain fatty acids (SCFAs) (40). In a previous study, an increase in solid feed intake was accompanied by an elevation in the relative abundance of *Syntrophococcus*, thereby enhancing the carbohydrate digestive capacity of early-weaned lambs (41). *Syntrophococcus* is involved in the utilization of non-fibrous carbohydrates and the production of acetic acid. *Butyricimonas* is a butyrate producer (42). Acetic acid and butyrate are SCFAs. In the gastrointestinal tract, SCFAs can enhance intestinal barrier integrity, regulate glucose and lipid metabolism and the immune system, modulate inflammatory responses, suppress pathogenic bacterial growth, and manage blood pressure (43, 44). l-carnosine supplementation increased the abundance of *Syntrophococcus* and *Butyricimonas*, which possibly improved the utilization efficiency of non-fibrous carbohydrates and SCFA levels. The abundances of *Escherichia-Shigella* and *Candidatus* Saccharimonas were significantly reduced in the L group versus the C group. *Escherichia-Shigella*, common intestinal pathogens, can cause intestinal diseases (e.g., enteritis, diarrhea, and dysentery) or extraintestinal conditions (e.g., urinary tract infections, meningitis, or sepsis) through attachment to epithelial cells and/or invasion of target host cells (45). *Candidatus* Saccharimonas, an opportunistic pathogen in the intestinal tract, is associated with gastrointestinal disorders and causes inflammatory diseases of the intestinal mucosa (46, 47). A previous study revealed a negative correlation between *Candidatus* Saccharimonas and production performance in geese (48). Our findings support that dietary l-carnosine supplementation in sheep increases the relative abundance of beneficial bacteria in the intestine, while decreasing that of pathogenic bacteria.

The serum concentrations of the metabolites l-histidine, d-apiose, and isodomedin were highly significantly upregulated in group L. d-Apiose is a branched pentose in the cell walls of higher plants (49) that has an antioxidant effect (50). The differential metabolites were significantly enriched in the histidine metabolic pathway, in which l-histidine was upregulated in group L. l-Histidine, as a precursor of histidine synthesis (51), increases the synthesis of ammonia, alanine, and glutamine in muscle and affects the anabolism of proteins (52). Isodomedin belongs to the class of diterpenes (53), which exhibit anti-inflammatory, antioxidant, and antimicrobial properties (54). Pyridine N-oxide glucuronide, a glucuronic acid-derivative, was highly significantly downregulated in group L. It has been shown that glucuronic acid is closely related to inflammation by acting on Toll-like receptor 4 and inducing pain in rats (55). We speculate that the addition of l-carnosine increased the antioxidant capacity in the fattening lambs to a certain extent. In summary, l-carnosine supplementation may have increased the antioxidant capacity and enhanced protein synthesis in the fattening lambs.

Comprehensive analysis of the microbiomes and non-target metabolomes unveiled the relationships between differential microbiota genera and differential metabolites. Notably, our results indicated a positive correlation between *Butyricimonas* and l-histidine and d-apiose. SCFAs, produced through microbial fermentation in the intestine, exhibit diverse physiological functions (56). *Butyricimonas* is a putative SCFA producer with potent anti-inflammatory and immunomodulatory effects (57). Amino acids serve as precursors for bacterial SCFA synthesis, demonstrating an interaction between microbial activity and the homeostasis of host amino acids and SCFAs (58). Stachydrine exerts potent anti-inflammatory action via the NF-κB signaling pathway (59) and prevents oxidative stress by increasing the activity of antioxidant enzymes such as superoxide dismutase (60). These findings suggest that *Butyricimonas* may enhance the nutrient conversion capacity and production performance by alleviating inflammatory responses and influencing metabolic processes associated with amino acids and carbohydrates. Phenylacetylglutamine is a metabolic byproduct of intestinal bacteria (41) and serves as a substitute for urea in the urea cycle to facilitate the excretion of nitrogenous waste from the body (61). *Pygmaiobacter* is the main butyric acid producer, and the correlation analysis showed that it was significantly positively correlated with phenylacetylglutamine, which may induce changes in intestinal microbes. We found a negative correlation between *Butyricimonas* and pyridine N-oxide glucuronide. Pyridine N-oxide glucuronide promotes inflammation. These findings corroborate that l-carnosine has anti-inflammatory properties. Comprehensive analysis of the microbiome and non-target metabolomes further showed that l-carnosine promoted weight gain in fattening sheep by exerting anti-inflammatory and increasing antioxidant property.

## 5 Conclusion

Dietary l-carnosine supplementation in fattening lambs was found to improve growth performance by positively influencing the intestinal microbiota and serum metabolites. Enhanced oxidative capacity, protein synthesis ability, and anti-inflammatory activity may be among the main mechanisms of l-carnosine in increasing weight gain during the fattening period. Therefore, l-carnosine shows promise for application in the sheep industry.

## Data Availability

The datasets presented in this study can be found in online repositories. The names of the repository/repositories and accession number(s) can be found in the article/Supplementary material.

## Footnotes

^1^ http://www.hmdb.ca/

^2^ https://metlin.scripps.edu/

^3^ https://www.ncbi.nlm.nih.gov/sra/PRJNA1175011

## Supplementary material

SUPPLEMENTARY FILE S1 Differences in serum metabolite profiles and changes in serum metabolites.
