# Supplementary material for: Effects of dietary l-carnosine supplementation on the growth, intestinal microbiota, and serum metabolome of fattening lambs
Source: Front Vet Sci. 2025 Jan 22;11:1525783. doi: 10.3389/fvets.2024.1525783 (PMC11795826; doi:10.3389/fvets.2024.1525783)
Supplement: SUPPLEMENTARY FILE S1 — Differences in serum metabolite profiles and changes in serum metabolites. [file Supplementary_file_1.docx]

**Additional file 1: Fig. S1A and S1B** **Difference of serum metabolite profiles of fattening sheep with different diets.**

**(S1A)** The OPLS-DA score plot for serum metabolites of L-carnosine group and control group. **(S1B)** the model overview showing high R2Y and Q2 in OPLS-DA of serum metabolites.
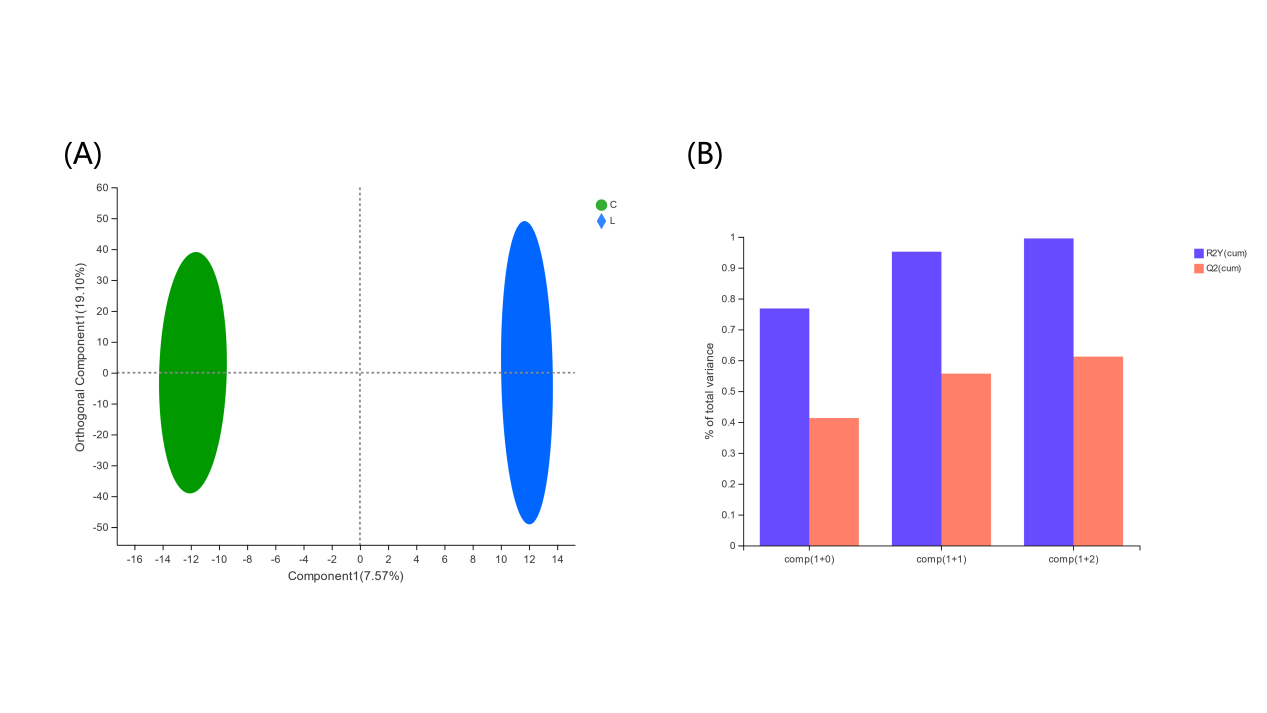


**Supplementary Table 1. VIP hierarchical clustering heatmap data table**

Changes in serum metabolites of fattening sheep after addition of l-carnosine. “up/down” indicate the increase/decrease in the metabolite level after adding l-carnosine to the diet.

| Metabolite | VIP_Oplsda | P_value | Regulate |
| --- | --- | --- | --- |
| Pyridine N-oxide glucuronide | 4.2263 | 4.58E-05 | down |
| Isodomedin | 3.6276 | 0.0001 | up |
| Clofenotane | 3.4196 | 0.0031 | down |
| 3,4,5-trihydroxy-6-[(2-hydroxyacetyl)oxy]oxane-2-carboxylic acid | 3.3541 | 0.0014 | down |
| 3-amino-2-naphthoic acid | 3.2799 | 0.0121 | down |
| (2-{[3-(3,4-dihydroxyphenyl)prop-2-enoyl]oxy}ethyl)trimethylazanium | 3.1382 | 0.0011 | down |
| Ethyl nitrite | 3.0927 | 0.0222 | down |
| Citreoviridinol A1 | 3.0713 | 0.0128 | up |
| GPCho(15:1/19:0) | 3.0618 | 0.0369 | up |
| Deoxyvasicinone | 2.9865 | 0.0011 | down |
| DL-Ornithino-L-alanine | 2.9374 | 0.0290 | up |
| GPCho(16:0/22:4) | 2.8911 | 0.0164 | up |
| N-(3-aminopropyl)-3-(3,4-dihydroxyphenyl)propanimidic acid | 2.8466 | 0.0216 | down |
| LysoPA(18:2(9Z,12Z)/0:0) | 2.8015 | 0.0029 | up |
| Zileuton O-glucuronide | 2.6947 | 0.0013 | up |
| Austalide J | 2.6903 | 0.0476 | up |
| Tyramine glucuronide | 2.6673 | 0.0148 | down |
| 4-hydroxy-5-[4-hydroxy-3-(sulfooxy)phenyl]pentanoic acid | 2.6538 | 0.0008 | up |
| Asparaginyl-Phenylalanine | 2.5453 | 0.0208 | down |
| 1-Pyrroline-5-carboxylic acid | 2.5421 | 0.0162 | down |
| L-Histidinol | 2.5374 | 0.0006 | up |
| Momordin Ie | 2.4473 | 0.0406 | up |
| PS(20:1(11Z)/0:0) | 2.4107 | 0.0022 | down |
| Asteltoxin | 2.3426 | 0.0496 | up |
| 3,4-DIDESMETHYL-5-DESHYDROXY-3'-ETHOXYSCLEROIN | 2.3052 | 0.0454 | up |
| Glucosamine 6-phosphate | 2.2772 | 0.0154 | down |
| D-Apiose | 2.2288 | 0.0001 | up |
| Aspartyl-Serine | 2.2229 | 0.0286 | down |
| Somatostatin | 2.2067 | 0.0136 | down |
| Tyrosyl-Histidine | 2.1792 | 0.0406 | down |
